# Critical Role of VCP/p97 in the Pathogenesis and Progression of Non-Small Cell Lung Carcinoma

**DOI:** 10.1371/journal.pone.0029073

**Published:** 2011-12-22

**Authors:** Christopher W. Valle, Taehong Min, Manish Bodas, Steven Mazur, Shahnaz Begum, Danni Tang, Neeraj Vij

**Affiliations:** 1 Johns Hopkins Physical Sciences in Oncology Center, The Johns Hopkins School of Medicine, Baltimore, Maryland, United States of America; 2 Department of Pediatric Respiratory Sciences, The Johns Hopkins School of Medicine, Baltimore, Maryland, United States of America; 3 Institute of NanoBiotechnology, The Johns Hopkins School of Medicine, Baltimore, Maryland, United States of America; 4 Department of Pathology, The Johns Hopkins School of Medicine, Baltimore, Maryland, United States of America; Virginia Commonwealth University, United States of America

## Abstract

**Background:**

Valosin-containing protein (VCP)/p97 is an AAA ATPase molecular chaperone that regulates vital cellular functions and protein-processing. A recent study indicated that VCP expression levels are correlated with prognosis and progression of non-small cell lung carcinoma (NSCLC). We not only verified these findings but also identified the specific role of VCP in NSCLC pathogenesis and progression.

**Methodology/Principal Findings:**

Our results show that VCP is significantly overexpressed in non-small cell lung carcinoma (NSCLC) as compared to normal tissues and cell lines (p<0.001). Moreover, we observed the corresponding accumulation of ubiquitinated-proteins in NSCLC cell lines and tissues as compared to the normal controls. VCP inhibition by si/shRNA or small-molecule (Eeyarestatin I, EerI) significantly (p<0.05, p<0.00007) suppressed H1299 proliferation and migration but induced (p<0.00001) apoptosis. Cell cycle analysis by flow cytometry verified this data and shows that VCP inhibition significantly (p<0.001, p<0.003) induced cell cycle arrest in the G0/G1 phases. We also found that VCP directly regulates p53 and NFκB protein levels as a potential mechanism to control tumor cell proliferation and progression. Finally, we evaluated the therapeutic potential of VCP inhibition and observed significantly reduced NSCLC tumor growth in both *in vitro* and xenograft murine (athymic-nude) models after EerI treatment (p<0.05).

**Conclusions/Significance:**

Thus, targeting VCP in NSCLC may provide a novel strategy to restore p53 and NFκB levels and ameliorate the growth and tumorigenicity, leading to improved clinical outcomes.

## Introduction

Valosin-containing protein (VCP)/p97 is an ATPase associated with various cellular activities (AAA) including the cell cycle, apoptosis, gene transcription, proteostasis, and the DNA damage response [1], [2], [3], [4]. VCP regulates these diverse functions *via* the ubiquitin-proteasome system (UPS). It is a molecular chaperone that controls retrograde translocation of ubiquitinated substrate proteins to the proteasome for their degradation [5].

VCP/p97 is associated with the various cellular pathologies and disease states including neurodegenerative disorders (Creutzfeldt-Jacob, Alzheimer's, and Parkinson's diseases) [6], [7], pulmonary conditions [8] and other protein misfolding disorders [9], [10]. Additionally, clinical studies have identified a correlation between elevated VCP expression and the progression, prognosis, and metastatic potential of esophageal carcinoma [11], colorectal carcinoma [12], and prostate cancer [13]. More recently, global genomic analysis of pancreatic cancer has confirmed VCP overexpression by **S**erial **a**nalysis of **g**ene **e**xpression (SAGE). Moreover, this study identified VCP as one of the few known recurrent amplicons at the DNA level associated with tumor metastasis [14]. Other studies have indicated the generalized role of UPS in regulating the metastatic potential of pancreatic cancer [15] and osteosarcoma [16] through the NFκB signaling pathway. The study on osteosarcoma [16] also indicates the UPS function of VCP as a regulator of NFκB mediated tumor metastasis.

Elevated VCP expression has also been found to *correlate* with the progression and clinical prognosis of non-small cell lung carcinoma (NSCLC) [17], which accounts for roughly 85% of all cases of lung cancer. Although lung cancer is the leading cause of cancer-related mortality in the United States (http://www.cancer.org/Research/CancerFactsFigures/index), the specific molecular mechanisms linking VCP expression with NSCLC pathogenesis and progression have not yet been identified. Thus, this study was designed to identify the molecular mechanism by which VCP regulates NSCLC pathogenesis and also evaluate the therapeutic potential of small-molecule targeting VCP dependent pathways.

Our data demonstrate that VCP inhibition controls NSCLC proliferation and progression by regulating tumor cell growth, migration, and apoptosis. We found that VCP inhibition induces G0/G1-phase cell cycle arrest of NSCLCs. We also identify here the role of VCP in controlling the protein levels of critical metastatic regulator- NFκB and tumor suppressor- p53 in NSCLC. Based on these results, we suggest that VCP regulates NSCLC tumor- genesis and metastasis *via* NFκB and p53 by a UPS-mediated mechanism. Moreover, we found that VCP inhibition by small molecule significantly (p<0.05) reduced NSCLC tumor growth in both *in vitro* and *in vivo* models. In summary, we identify and verify the therapeutic potential of a novel molecular strategy targeting VCP to ameliorate the NSCLC growth and tumorigenicity.

## Materials and Methods

### Ethics Statement

All animal experiments were carried out in accordance with the Johns Hopkins University (JHU) Animal Care and Use Committee (ACUC) approved protocol (MO11M271). The human lung tissue sections were collected from Lung Tissue Research Consortium (LTRC) and JHU pathology core in a blinded manner (without disclosing the subject's name and information) and were approved by the Institutional Review Board (IRB), Johns Hopkins University as exempt (not a human subject research).

### Culture Conditions, Transfections and Treatments

The human bronchial epithelial cells, HBE and Beas2b (obtained from autopsy of non-cancerous individual, ATCC) were cultured in MEM and DMEM/F12 medium, respectively. While human non-small cell lung carcinoma cell lines (adeno-NSCLC, H1299;p53- and H1944;p53+) and the alveolar adenocarcinoma NSCLC (A549; p53+) were cultured in RPMI 1640 and DMEM medium, respectively. The media were supplemented with 10% Fetal Bovine Serum (FBS) and 1% Penicillin, Streptomycin, Amphotericin B (PSA) from Invitrogen and cells were grown in an incubator at 37°C with 5% CO_2_. The cells were transiently transfected with Lipofectamine 2000 (Invitrogen) following the manufacturer's instructions. To inhibit VCP expression in H1299/H1944 cells by RNA interference, short hairpin RNAs (shRNAs) targeting the sequence of VCP cloned into the pSHAG-MAGIC2 (pSM2) vector (Open Biosystems)were used. The VCP shRNA (0.2 µg for 96-well; 1.6 µg for 12-well; 4 µg for 6-well) knock down efficiencies were compared to empty vector (at same concentration as VCPshRNA) transfected control cells. In addition, Dharmacon ON-TARGET*plus* SMARTpool of VCP siRNA (Thermo Scientific; 100 nm) was used to verify VCP inhibition data as compared to the control siRNA (100 nm). While, VCP function was inhibited by small molecule VCP inhibitor Eeyarestatin I (EerI) [18] at a final concentration of 10 µM for 24 hrs and compared to DMSO control vehicle. The pcDNA3.1-VCP-myc and control plasmids were transfected as above for over expression experiments.

### Immunofluorescence Microscopy of Clinical Biopsies from NSCLC Patients-

Formalin-fixed, paraffin-embedded longitudinal tissue sections from adeno- and squamous- NSCLC human subjects were used to evaluate the localization and expression of VCP and ubiquitin in tumor and surrounding control tissue. Tissues were immunostained with primary antibodies (1∶200 dilution) for VCP (rabbit polyclonal, Santa Cruz Biotechnology [SCBT]) and ubiquitin (mouse monoclonal, SCBT) followed by secondary antibodies (1∶200 dilution) using our previously described protocol [19]. The secondary antibodies used were goat anti-rabbit IgG FITC (SCBT) and donkey anti-mouse Dylight 594 (Jackson ImmunoResearch). Nuclei were detected by Hoechst (Invitrogen) staining. Images were captured by Axiovert 200 Carl Zeis Fluorescence microscope using the Zeiss Axiocam HRC camera and Axiovision software.

### Immunoblotting and Immunoprecipitation

All immunoblotting (IB) and immunoprecipitation (IP) experiments were performed as described previously [19]. Briefly, total protein extracts were prepared from the control and NSCLC cell lines or transfected cells using 1× Mammalian protein extraction reagent (M-PER, Pierce) supplemented with protease inhibitor cocktail (Sigma) and 5 mM EDTA (Sigma). Protein extracts were then resolved by SDS-PAGE followed by IB with antibodies against VCP (1∶500, SCBT), p53 (1∶500, SCBT), NFκB (1∶500, SCBT), ubiquitin (1∶1000, SCBT), Nrf2 (1∶500, SCBT), SIRT-1 (1∶250, SCBT), and β-actin (equal loading control, 1∶2500, Sigma). Proteins were detected with the HyGlo™ Chemiluminescent HRP Antibody Detection Reagent and exposed to HyBlot CL™ Autoradiography film (Denville Scientific). To examine the binding of p53 to VCP, we incubated two aliquots of 500 µg/ml total protein extract with 50 µL of protein A/G PLUS-agarose beads (SCBT) for 3 hours at 4°C. After pre-clearing, 1 µg of mouse VCP antibody was added to one tube, and pre-immune serum was added to the other tube as a negative control. After 1 hour, the agarose beads (50 µL) were added to each tube followed by overnight incubation at 4°C. The beads were washed three times with ice cold PBS followed by heating at 100°C for 2–3 minutes in loading dye containing β-mercaptoethanol. The IP's were resolved by SDS-PAGE and immunoblotted using rabbit p53 antibody (as above) to identify the protein-protein interaction.

### Cell Proliferation Assay

Cell growth was quantified by MTT (3-(4,5-Dimethylthiazol-2-yl)-2,5-diphenyltetrazolium bromide) assay as described previously [20]. Briefly, H1299 cells were seeded onto 96-well plates at a concentration of 2500 cells/well, followed either by treatment with EerI (10 µM) or DMSO (vehicle), or transfection with VCP- or control-siRNA. After 22- (inhibitor) or 14- (transfected) hours of incubation, 10 µl of 5 mg/ml MTT reagent (Cell Titer 96® AQ_ueous_ One Solution, Promega) was added to each well. The cells incubated for at least 2 hours with MTT followed by analysis of absorbance (490 nm) using a VERSAmax microplate reader (Molecular Devices) at the indicated time points.

### Flow Cytometry-Based Cell-Cycle Analysis-

H1299 cells (1×10^6^) were treated with EerI (10 µM) and DMSO (vehicle) for 24 hours or were transfected with VCPshRNA and pSM2 control vector for 48 hours. Cells were rinsed with PBS and subsequently fixed in ice-cold ethanol (70% v/v) overnight. The fixed cells were then washed twice with by PBS followed by re-suspension in a staining solution containing 10 µg/ml propidium iodide (PI; Sigma), 20 µg/ml RNAse A (Sigma), and BSA (0.1%w/v). After a 1-hour incubation at room temperature, DNA content was determined using a FACScan flow cytometer (BD Biosciences) as described previously [21]. Cell-cycle distribution was analyzed using Cell Quest software (BD Biosciences). Red fluorescence (582±42 nm) was evaluated on a linear scale after pulse-width analysis was used to exclude cell doublets and aggregates from analysis. Cells with DNA content between 2 N and 4 N were then designated as being in the G0-/G1-, S-, or G2/M-phase of the cell cycle, and cell populations in each phase were determined.

### Caspase-3/7 Enzyme Assay-

Apoptotic activity was also quantified using the Caspase-3/7 Glo Assay Kit (Promega), as described previously [22]. Briefly, H1299 cells were seeded onto a 96-well, black-bottomed plate at a concentration of 2500cells/well in 100 µl culture medium and allowed to adhere overnight. Cells were then treated with EerI (10 µM) or DMSO (vehicle) or transfected with VCP shRNA or control plasmid for indicated time points as above. Subsequently, 100 µl of the freshly prepared caspase-3/7 reagent was added, followed by 1 hour incubation at room temperature. Luminescence was quantified using a Spectramax microplate reader (Molecular Devices, excitation- 498 nm and emission- 521 nm).

### Cell Scratch Assay-

H1299 cells were transfected with VCPshRNA and pSM2 control vector so as to form a fully confluent monolayer on a 12-well plate. After 36 hours, a p10 pipette tip was used to create a ∼2 cm scratch on the monolayer. Cells were washed twice with PBS to remove all debris and then washed with RPMI 1640 media. Alternatively, a scratch was created on a fully confluent monolayer, followed by PBS washes, and treatment with either EerI (10 µM) or DMSO (vehicle). The cells were then allowed to proliferate and migrate for the following 24 hours. Images were collected 8, 12 and 24 hours after the scratch (assigned time 0 hours) using a Nikon Eclipse TS100 inverted light microscope with a 10× phase objective and Infinity Capture software. Changes in the width of the scratches over time were later analyzed using Adobe Photoshop software.

### Transwell Invasion Assay

H1299 cells were transfected with control pSM2 vector or VCPshRNA-2 plasmid for 24 hours. Cells were then trypsinized and plated (5000 cells) on Matrigel (BD Biosciences, 200 µg Matrigel in 400 µl serum-free medium) coated transwell inserts (0.4 µm pores, Millipore). After 24 hrs, cells that had migrated through and adhered to the bottom surface of the insert were stained with Trypan Blue (Life Technologies) and counted under a light microscope.

### *In vitro* Tumor Cell Growth Model-

RPMI-1640 medium supplemented with 10% fetal bovine serum and 0.6% of agarose (Research Products International) was prepared and poured into each well of a 6-well plate as a base agar layer. H1299 cells (4×10^5^) were then suspended in 0.3% molten agarose and layered on top of the solidified base agar in each dish. These dishes were placed at 37°C and 5% CO_2_ in a cell culture incubator for 1 hour to allow the top layer to solidify. Fresh RPMI-1640 media containing either EerI (10 µM) or DMSO (vehicle) was then layered on top to treat the cells (n = 3) and prevent the agarose from drying. The plates were returned to the cell culture incubator at 37°C and 5% CO_2_ for 48-hours. Images of each well were captured and densitometry analysis was performed using Image J software to quantify statistical changes in tumor cell growth.

### Xenograft Model

All animal experiments were carried out in accordance with the Johns Hopkins University Animal Care and Use Committee (ACUC) approved protocols. We used age-, weight-and sex-matched (8–10 weeks old) athymic nude mice (NCI Animal Production Program). All mice were housed in controlled environment and pathogen-free conditions. Briefly, H1299 cells (8×10^6^) were suspended in 100 µl PBS media containing Matrigel (1∶1 v/v, BD BioSciences) and subsequently injected subcutaneously (s.c.) into the sub-scapular region of the mice. After two days, mice were randomized into treatment and control groups (n = 5) and given either 50 µg EerI in 100 µl PBS or appropriate DMSO vehicle (0.01% v/v) by s.c. injection directly into the tumor. Drug treatment was repeated on day-6. Tumor size was determined by measurement of the largest and perpendicular diameters every week till week-6 followed by terminal euthanasia following procedures of our ACUC approved protocol. Tumor volume (V) was calculated according to the formula *V = 0.52 ab^2^*, where “*a*” is the largest superficial diameter and “*b*” is the diameter orthogonal to “*a*”.

### Statistical and Image Analysis-

Representative data is shown as the mean ± SEM of at least three experiments. Variations in data between the different groups were tested by a two-tailed unpaired t-test. A p-value less than 0.05 was considered significant. Changes in protein expression were analyzed by densitometry using the Image J 1.38× software (NIH, Bethesda, MD).

## Results

### Proteostasis-imbalance Induces VCP Expression in Adeno- and Squamous- NSCLC

**Figure 1:**
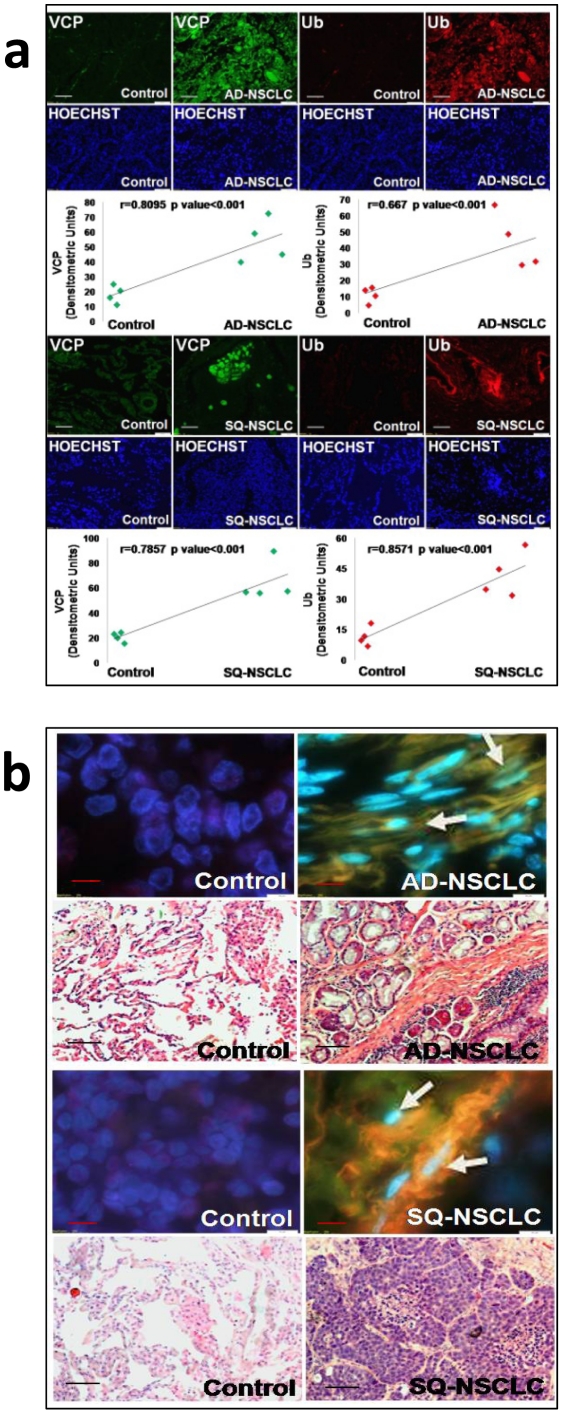
Elevated VCP protein levels and proteostasis-imbalance in NSCLC. (**a**) We examined the expression of VCP in adeno(AD)- and squamous(SQ)-NSCLSC as compared to normal tissue and observed a significant increase (p<0.001) in VCP expression and ubiquitin accumulation in both NSCLCs (n = 4, White bar = 100 µm). (**b**) We confirmed that VCP and ubiquitinated proteins are co-localized in peri-nuclear aggregates of these tissues (n = 4, Black bar = 100 µm & Red bar = 10 µm). *Elevated VCP protein expression and ubiquitin accumulation in NSCLC indicates proteostasis-imbalance.*

A previous clinical study has indicated that elevated VCP protein expression is a prognosticator for overall survival in both early and advanced pathologic stages (pT) of non-small cell lung carcinoma (NSCLC) [17]. To substantiate these findings, we first analyzed human NSCLC tissue samples for changes in VCP protein levels by immunofluorescence microscopy. Our data not only confirm significantly elevated VCP protein expression but also demonstrate the accumulation of ubiquitinated proteins (p<0.001) in both adeno(AD)- and squamous(SQ)-NSCLC as compared to the surrounding non-neoplastic control lung tissue (Figure 1a). We anticipate that accumulation of ubiquitinated proteins is due to the reduced proteasomal activity as reported earlier for other cancer tissues [23]. Moreover, we found that VCP and ubiquitin are co-localized in peri-nuclear aggregates in these tissues (Figure 1b, representative areas). These findings, suggest a causative role of proteostasis deficiency in NSCLC indicating that the accumulation of ubiquitinated proteins resulting from proteostasis-imbalance [8], [10] induce VCP expression in NSCLC. Although elevated VCP expression has been correlated to probability of survival for NSCLC patients [17], its functional role in NSCLC is not apparent.

### VCP Regulates NSCLC Proliferation and Apoptosis

**Figure 2:**
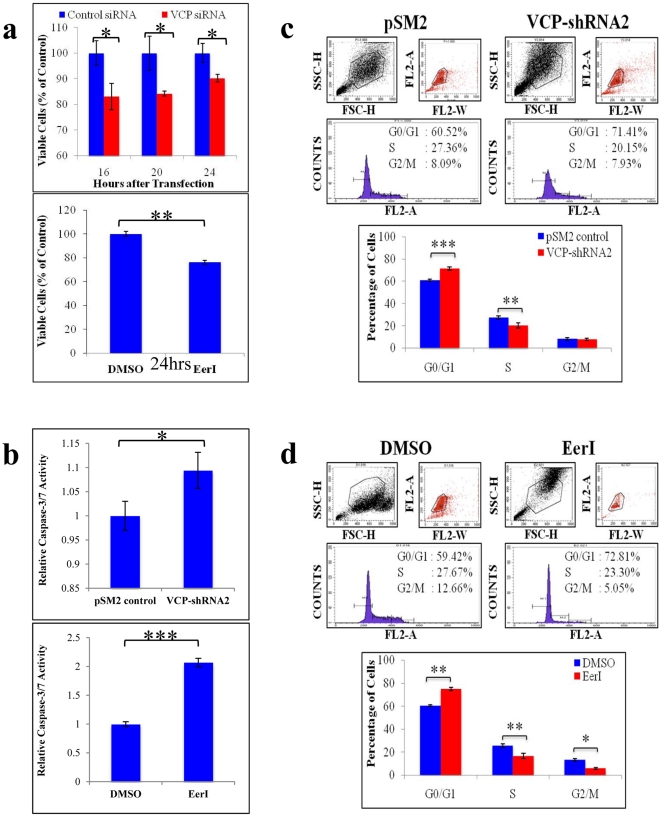
VCP regulates NSCLC proliferation, apoptosis and cell division. (**a**) H1299 cells were seeded on a 96-well plate and either transfected with pSM2 control vector or VCP-shRNA (for 16 hrs) or treated with EerI (10 µM) or DMSO (vehicle) for 24 hrs. Promega Aq_ueous_ One MTS reagent was added to each well, 2 hours before stopping the experiment and VERSAmax microplate reader was used to quantify the MTS activity (n = 4) at indicated time points. Our data shows a significant (p<0.01) decrease in cell proliferation by VCP inhibition. (**b**) H1299 cells were seeded on a black-bottomed 96-well plate and either transfected with pSM2 control vector or VCP-shRNA or treated with EerI or DMSO. After 48 (VCP-shRNA) or 24 (EerI) hours, caspase-3/7 activity was quantified using the luminescence substrate (Promega). Our data shows an increase in caspase-3/7 activity after transient VCP knockdown (* p<0.05) and a very significant increase (two-fold) in caspase-3/7 activity after treatment with functional inhibitor of VCP, EerI (*** p<0.00001). Next, (**c**) H1299 cells were either transfected with VCP-shRNA2 and pSM2 control vector or (**d**) treated with EerI or DMSO. Cells were then fixed in 70% ethanol followed by propidium iodide (10 µg/ml) staining for 1 hour. The profiles of DNA content (measured by a FACScan flow cytometer) for cell-cycle distributions are shown. Our data, summarized in the bar graph (bottom of each panel), shows that VCP knockdown induced G0/G1 arrest (*** p<0.001) and reduced the number of cells undergoing DNA replication (S-phase; ** p<0.01). Inhibition of VCP's function by EerI not only induced G0/G1 arrest (** p<0.01) and decreased DNA replication (** p<0.01) but also significantly reduced the number of cells undergoing mitosis (G2/M-phase; * p<0.05). *VCP inhibition controls the tumorigenic capacity of NSCLC by slowing proliferation and division while simultaneously inducing apoptosis.*

To investigate the role of VCP in the pathogenesis and progression of NSCLC, we inhibited VCP expression or function and quantified the changes in NSCLC proliferation, apoptosis, and cell cycle. First, we used cell proliferation assays to quantify changes in proliferation induced by VCP inhibition. Our data shows that inhibition of VCP protein expression by VCP siRNA significantly (p<0.05) reduced H1299 proliferation as early as 16 hours of transfection (Figure 2a, top panel). Similarly, treatment with a functional inhibitor of VCP, Eeyarestatin I (EerI, 24 hrs), significantly (p<0.01) reduced H1299 proliferation (24.0%) as compared to the vehicle-treated control (Figure 2a, bottom panel).

We then examined the effect of VCP inhibition on apoptosis of H1299 cells by a TUNEL assay (Methods S1). We observed an increase in the number of TUNEL-positive (green, apoptotic) cells by shRNA mediated VCP inhibition as compared to controls transfected with empty pSM2 vector (Figure S1, left panel, representative images). The TUNEL results were quantified by randomly selecting and analyzing 100 cells per sample (n = 3) and then calculating the average number of apoptotic cells. Our data (Figure S1, right panel) shows a significant 6-fold increase (p<0.00001) in the number of TUNEL-positive cells after VCP knockdown as compared to the controls. To confirm these findings, we quantified the changes in caspase-3/7 enzymatic activity after VCP inhibition. Caspase-3/7 are key mediators of apoptosis, and their activity offers a highly quantitative means for measuring cellular apoptosis. Our results indicate that knockdown of VCP expression by VCP-shRNA2 significantly (Figure 2b, top panel, p<0.05) increased caspase activity compared to the controls. Moreover, functional disruption of VCP-mediated proteasomal degradation by EerI caused a significant (∼2-fold, Figure 2b, bottom panel, p<0.00001) increase in caspase-3/7 activity relative to vehicle-treated controls. Our findings suggested that VCP regulates NSCLC proliferation and apoptosis and indicate its critical role in regulating the tumor cell cycle.

### VCP Regulates Cell Division of NSCLC

Next, we investigated the role of VCP in regulating the NSCLC cell cycle. For these experiments, we inhibited VCP expression or function in H1299 cells as above to quantify changes in cell cycle distribution by propidium iodide staining and flow cytometry. Transient transfection with VCP-shRNA2 (Figure 2c) significantly (p<0.001) induced a G0/G1-phase arrest (∼11%). This is confirmed by the significant (p<0.01) decrease in the number of cells in the S- phase (∼7%) as compared to the control. Our data demonstrates a more substantial change after selective inhibition of the ERAD (endoplasmic reticulum-associated degradation) function of VCP by EerI treatment (Figure 2d). We found that EerI treatment induced G0/G1-phase arrest (∼13%, p<0.01) with corresponding decreases in the relative number of cells in the S- (∼4%, p<0.05) and G2/M-phases (7%, p<0.01) as compared to the vehicle treated control. Although in both cases VCP inhibition triggered G0/G1-phase growth arrest and reduced the number of cells undergoing DNA replication in the S-phase, only inhibition of VCP-mediated proteasomal degradation by EerI reduced the number of cells actively undergoing mitosis in the G2/M-phase. Our data demonstrates the critical role of VCP in regulating tumor cell cycle, suggesting its involvement in NSCLC progression and invasion.

### VCP Mediates NSCLC Migration, Invasion and Tumor Growth

**Figure 3:**
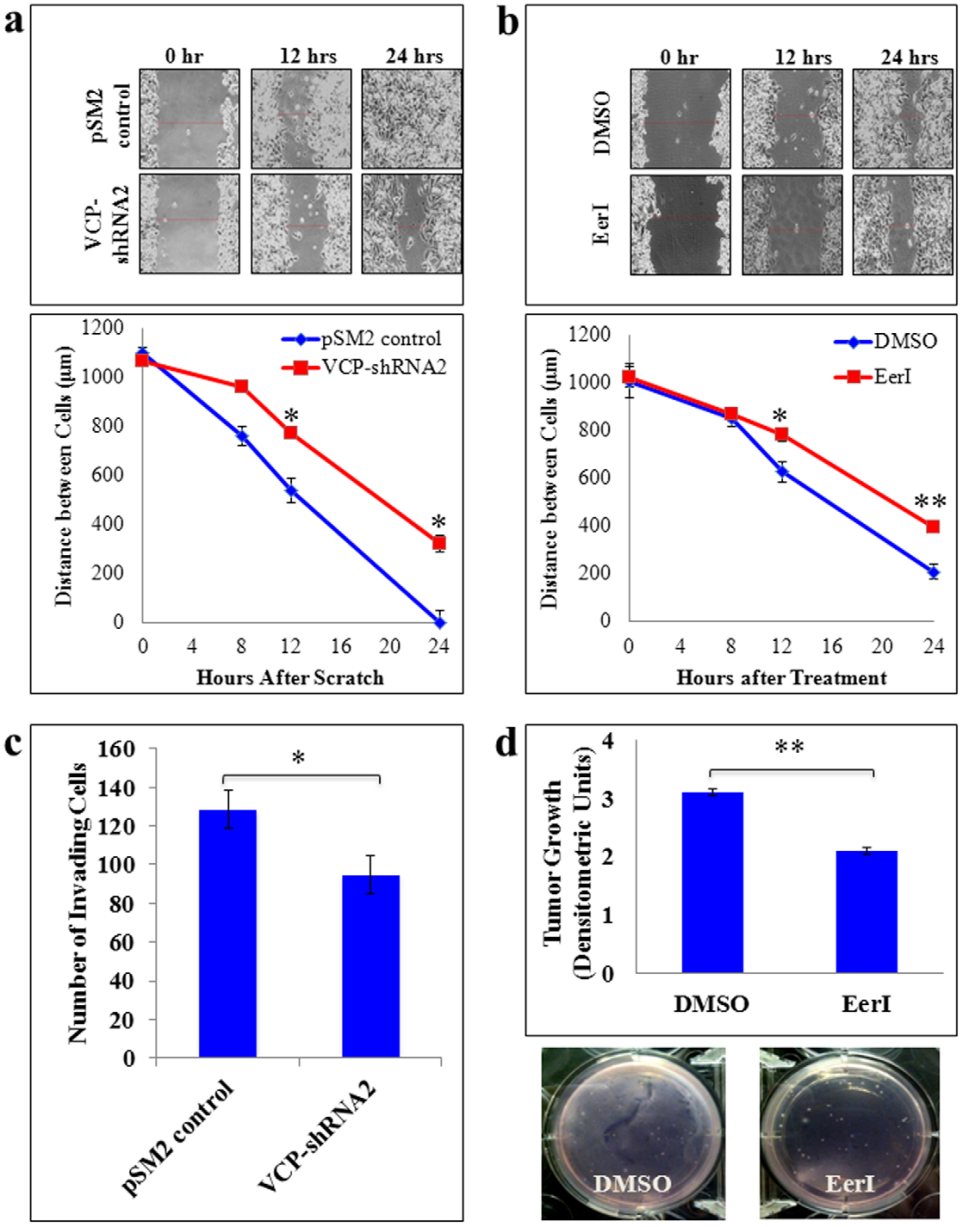
VCP inhibition retards the migration, invasion and growth of NSCLC. H1299 cells were either (**a**) transfected with pSM2 control vector or VCP-shRNA or (**b**) treated with EerI or DMSO, followed by scratch assay to quantify changes in cell migration. Briefly, a scratch was made across the center of the confluent monolayer using a 10 µl pipette tip. The cells were allowed to migrate and were monitored at the indicated time points using a Nikon light microscope and Infinity Capture Software. The representative images show that both VCP knockdown and inhibition visibly slowed H1299 migration into the scratch by 12 and 24 hours (top panels). Changes in the average width of the scratch were calculated using Infinity Analyze Software (mean ± SEM; n = 3–4). Quantitative analysis show that both VCP knockdown and functional inhibition significantly retarded cell migration at 12 and 24 hours (bottom panels, * p<0.05; ** p<0.0005). (**c**) H1299 cells were transfected with pSM2 or VCPshRNA2 for 24 hours and transferred to transwell inserts (BD, 0.4 µm pores) coated with 200 µg Matrigel basement matrix for additional incubation for 24 hours. Following incubation, cells that had migrated to the bottom of the membrane were stained with trypan blue solution and the central field of each insert was visualized using a Nikon light microscope (n = 4, mean ± SEM given). The data shows that VCP inhibition significantly reduced the number of invading cells as compared to the controls. (**d**) H1299 cells (4.0×10^5^ cells/well) were suspended in 2 mL of serum-containing medium with agarose (0.3%) and immediately plated over a layer of solidified agarose (2-mL, 0.6%) on a 6-well plate (n = 3). No cells (Blank) and untreated H1299 cells were used as controls. Fresh media containing either EerI (10 µM) or DMSO (vehicle) was added on top. After 48 hrs, the images were captured and analyzed using the Adobe Photoshop Software. Representative images and densitometry data (mean ± SEM) shows the significant decrease in number of colonies and H1299 cell invasion in agarose by EerI treatment as compared to the DMSO treated controls. *VCP mediates NSCLC migration, invasion and growth that suggest its critical role in tumor cell progression and metastasis.*

VCP has been shown to regulate NFκB signaling, which is critical for the metastasis of osteosarcoma cell line [16]. Hence, we anticipated that VCP might play a critical role in NSCLC migration, invasion, and metastasis. We tested this hypothesis by first quantifying changes in NSCLC migration and invasion after VCP knockdown or inhibition as described above. Knockdown of VCP expression (Figure 3a, representative images) reduced migration and invasion of H1299 cells into the scratch as compared to the controls. The distances remaining between the edges of the wounds (n = 3) were then quantified using Infinity Analyze software (Figure 3a, bottom panel). Quantification data confirms that VCP inhibition significantly (p<0.05) delayed the migration and invasion of the H1299 cells into the scratch as shown after 12 and 24 hours relative to the controls. We observed the same result with functional inhibitor of VCP, EerI (Figure 3b), further confirming the critical role of VCP in regulating NSCLC migration and invasion.

To verify these findings, we quantified the effect of VCP inhibition on NSCLC migration and invasion by transwell invasion assay of H1299 cells. H1299 cells were transfected with VCP-shRNA2 or pSM2 for 24 hours as above, followed by trypsinization and plating of 5000 cells in serum-free medium on a transwell insert coated with Matrigel. After 24 hours, we stained (Trypan Blue Dye) cells that had invaded through the Matrigel to the bottom of the membrane and counted the total number of cells in a 10× magnification field for all replicates. Our results show that VCP inhibition significantly (p<0.05) reduced the number of invading cells by ∼26% as compared to controls (Figure 3c). We also identified the critical role of elevated VCP expression in promoting tumor growth using the *in vitro* tumor growth model (described in the Materials and Methods). Briefly, H1299 cells (4×10^5^) were seeded into each well of a six-well plate (n = 3) containing a matrix composed of two layers of agarose [0.6% (bottom) and 0.3% (top)]. Our data shows that treatment of these cells with EerI significantly reduced *in vitro* tumor cell growth (Figure 3d, p<0.01). The data clearly shows the overall reduction in tumor cell growth (Figure 3d, representative images). Our results demonstrate the critical role of VCP in promoting NSCLC migration and invasion, implicating VCP in tumor metastasis.

### VCP Regulates Tumor Suppressor p53 and NFκB Protein Levels

**Figure 4:**
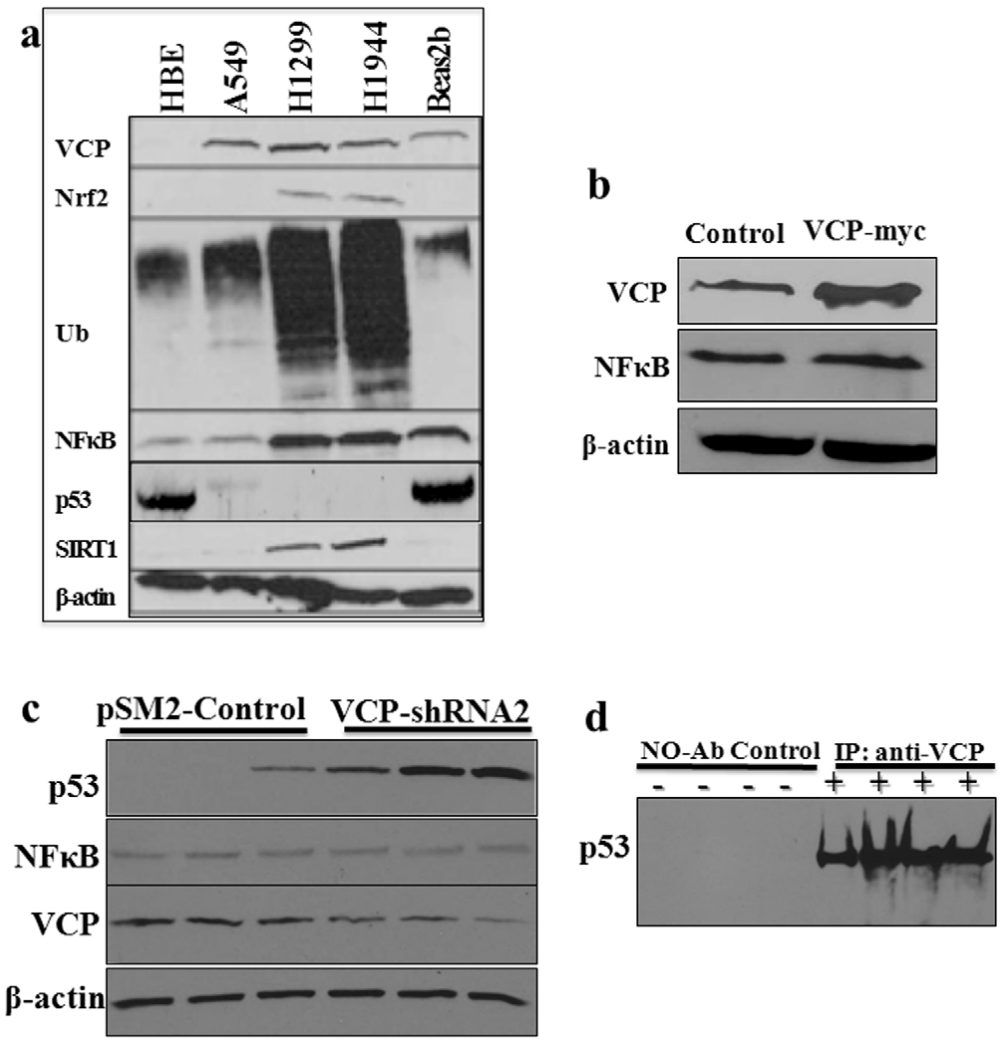
VCP regulates protein levels of p53, NFκB and other cancer-related proteins. (**a**) We observed the accumulation of ubiquitinated proteins, induction of VCP, Nrf2, SIRT1 (Sirtuin 1, stress regulator), and NFκB, and suppression of p53 in NSCLC cell lines (H1299 and H1944) as compared to control (Beas2b and HBE) cells indicating towards proteostasis-imbalance. β-actin antibody was used as an equal-loading control. NFκB, p53, Nrf2 and SIRT1 are known to be involved in tumor metastasis, proliferation, apoptosis and inflammatory-oxidative stress responses. (**b**) VCP overexpression (VCP-myc) in H1299 cells shows an increase in NFκB protein levels. (**c**) H1944 cells transfected with VCP shRNA (lane 4–6) show a significant increase in p53 protein levels and decrease in NFκB, as compared to pSM2 control (lanes 1–3). VCP immunoblot verifies VCP inhibition while β-actin shows equal loading. (**d**) Co-immunoprecipitation (IP) of p53 with VCP antibody verifies the novel protein-protein interaction. IP lacking anti-VCP antibody was used as a negative control. *Regulation of oncogene and tumor suppressor by VCP provides a potential mechanism for its critical role in tumor progression and metastasis.*

Next, we identified the molecular mechanism by which VCP induces NSCLC pathogenesis and metastasis. We used NSCLC (A549, H1299, and H1944) and non-neoplastic control (HBE and Beas2b) cells to identify changes in expression levels of VCP, ubiquitin, and tumor associated proteins including tumor suppressor p53 [24], [25], NFκB [16], nuclear factor E2-related factor 2 (Nrf2) [26], and sirtuin1 (SIRT1) [27]. Our data verifies significantly elevated VCP expression and the accumulation of ubiquitinated proteins (aggregates) in NSCLC (Figure 4a) as compared to the control cells, in accordance with our findings from the lung tumor tissues (Figure 1). Moreover, we identify here a correlation between VCP overexpression (highest expression) and induction of tumor associated proteins- NFκB, Nrf2 and SIRT1, and down regulation of tumor suppressor-p53 in NSCLC (H1299&H1944) cell lines (Figure 4a), suggesting the critical role of VCP-mediated proteasomal degradation in tumor cell proliferation and metastasis.

VCP has been shown to regulate NFκB suggesting its role in tumor metastasis [16]. We anticipated that a similar mechanism was responsible for the pro-metastatic function of VCP in NSCLC as it is known to regulate the NFκB protein levels by mediating the proteasomal degradation of its endogenous inhibitor, IκB [16]. To verify this, we overexpressed VCP in H1299 cells and observed changes in the protein levels of the critical metastatic mediator, NFκB. We observed a corresponding upregulation of NFκB as compared to controls (Figure 4b), confirming our hypothesis that VCP mediates NSCLC metastasis by regulating the NFκB signaling pathway. Next, we further investigated whether VCP may also regulate the levels of critical tumor suprresor-p53 by a similar mechanism. We used H1944 cells for this experiment as H1299 cell line is p53 deficient [28], [29]. We observed that inhibition of VCP expression levels in H1944 cells significantly upregulates tumor suppressor p53 by VCP inhibition (Figure 4c). We also verified the decrease in NFκB by VCP inhibition. These results suggest that VCP may regulate p53-NFκB protein levels by mediating its proteasomal degradation of p53 and IκB, an endogenous inhibitor of NFκB. To substantiate this finding, we used immunoprecipitation to determine whether p53 and VCP interact in NSCLC cells. Our results demonstrate a novel VCP-p53 interaction (Figure 4d) in H1944 cells, suggesting that VCP mediates proteasomal degradation of p53, as previously shown for IκB [30], [31]. To summarize, we demonstrated that VCP regulates NFκB and p53 protein levels as a potential mechanism to control NSCLC pathogenesis and progression.

### VCP Inhibition Controls Tumor Growth in NSCLC-Xenograft Model

**Figure 5:**
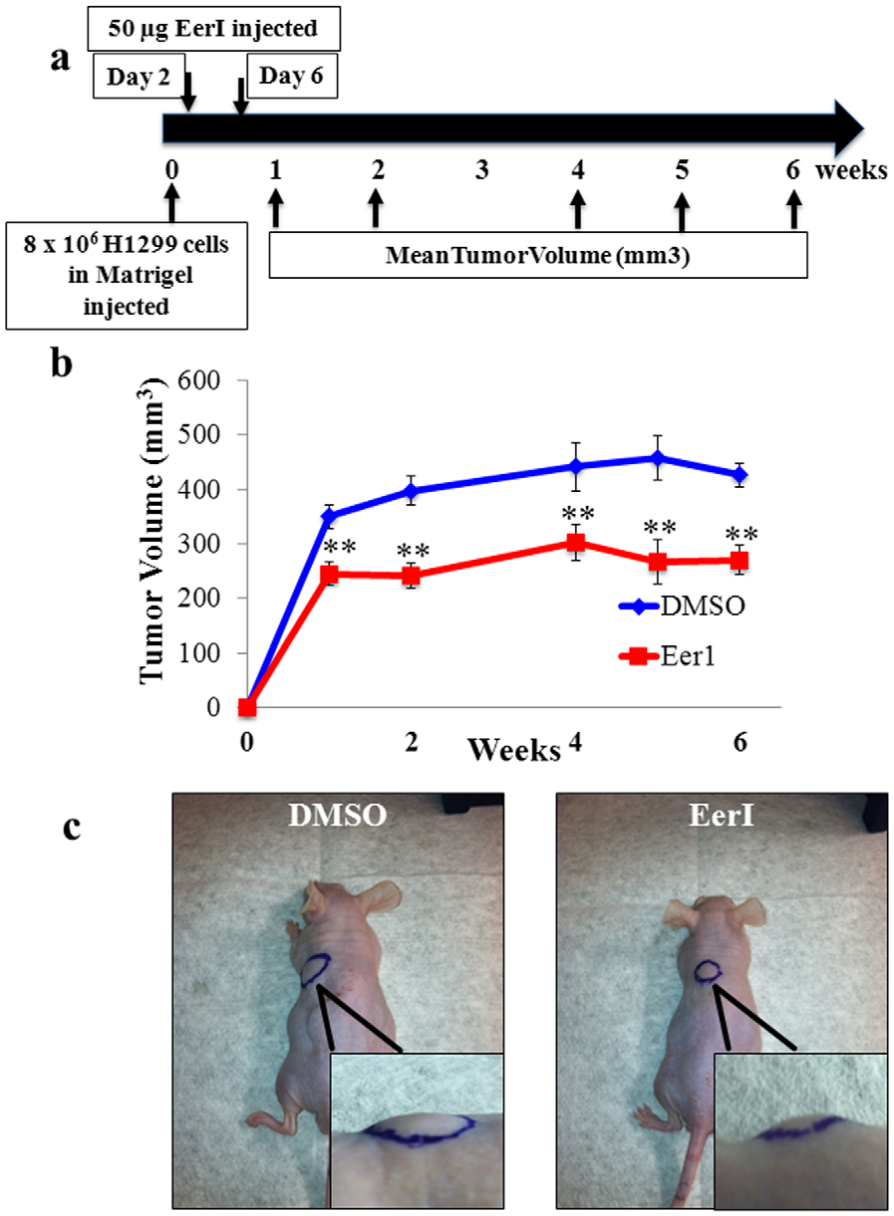
Inhibition of VCP's function by EerI reduces tumor growth in NSCLC-xenograft model. (**a**) Tumors were established in athymic nude mice by subcutaneous (s.c.) injection of H1299 (8×10^6^) cells complexed with Matrigel into the upper left flank. Mice were randomized into two groups (n = 5) and treated with EerI (50 µg, 2 doses at indicated times as on the scale) or DMSO vehicle. (**b**) Primary tumor growth was measured volumetrically over time and statistical analysis of data is shown as mean ± SEM. EerI treatment significantly reduced tumor volume as compared to the DMSO vehicle control ** p<0.01). (**c**) Representative images captured on Day 8 illustrate the difference in tumor volumes between the two groups. *VCP can be therapeutically targeted using EerI or other potent VCP-inhibitor to control NCLSC tumor growth and/or progression.*

We established NSCLC tumors in the subscapular region of athymic nude mice by subcutaneous (s.c.) injection of H1299 cells (8×10^6^) complexed with Matrigel. The mice were treated with functional inhibitor of VCP, EerI (10 µM or DMSO vehicle control) at indicated times on the scale (Figure 5a) and tumor volume was monitored every week (n = 5). Our data shows that EerI treatment significantly reduced tumor growth as compared to the DMSO vehicle control (Figure 5b & c). We observed consistent and significant reduction in primary tumor volume in EerI-treatment group (Figure 5b, p<0.05). This finding demonstrate the therapeutic potential of VCP inhibitor(s) in controlling NSCLC pathogenesis and progression; we are currently standardizing the dose and selective delivery of a novel VCP inhibitor based on its ability to control NSCLC progression and metastasis.

## Discussion

**Figure 6:**
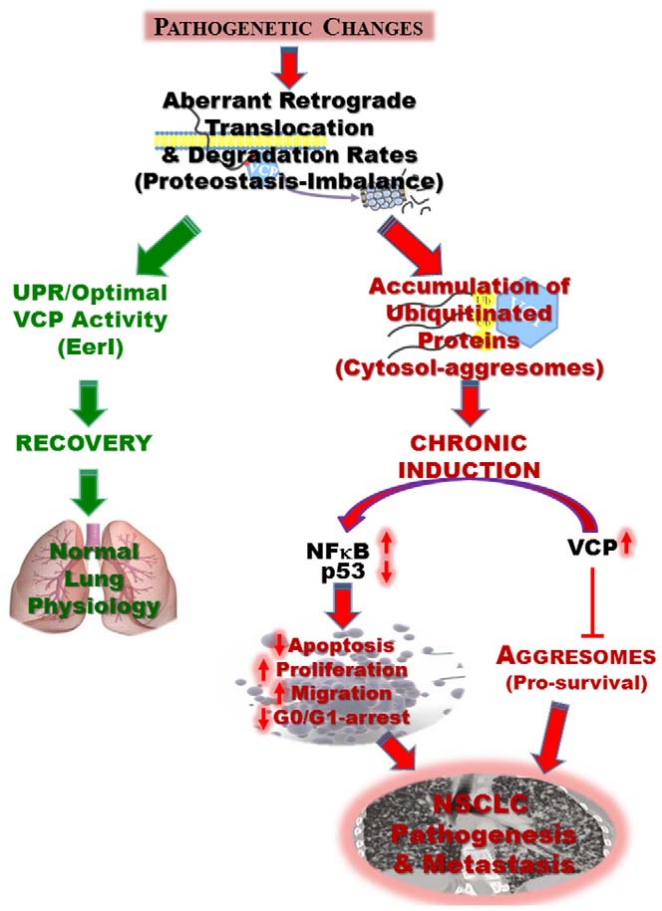
Schematic summarizing the functional role of VCP in NSCLC pathogenesis and progression. Our data suggests that elevated VCP expression and subsequent accumulation of ubiquitinated proteins (proteostasis-imbalance) de-regulates NSCLC proliferation, cell cycle, apoptosis and migration *via* NFκB and p53 pathways resulting in increased tumor- genesis and metastasis. We predict that increased retrograde translocation rates results in the accumulation of ubiquitinated aggregates that leads to chronic activation of VCP. This chronic VCP induction may lead to the pathological expression of key cancer-related proteins, including inhibition of tumor suppressor p53 and activation of key metastasis-protein NFκB, as seen in our study. *We propose that inhibition of VCP expression and/or function may control the progression and aggressiveness of NSCLC by rescuing vital cellular pathways from pathogenic activation.*

VCP is overexpressed in many solid tumors, including prostate and pancreatic cancers [13], [32], esophageal and colorectal carcinomas [11], [12], osteosarcoma [16]. Recent studies have also indicated that VCP expression may be an independent prognostic factor for overall survival in non-small cell lung carcinoma (NSCLC) [17], [33]. We verified elevated VCP protein expression not only in human adeno(AD)- and squamous(SQ)-NSCLC tissues (Figure 1) but also in the NSCLC cell lines A549, H1229 and H1944 (Figure 4a) as compared to non-cancer, HBE (>5-fold) or Beas2b (≥2-fold) cell lines. In addition to genetic changes, VCP expression can also be influenced by cigarette smoke exposure and/or age [34], [35] (pathogenetic changes, Figure 6). The elevated VCP expression has several pathogenic consequences, it is a major component of retrograde translocation machinery that pulls out ubiquitinated protein from ER membrane to proteasome for degradation [9]. The increased VCP retrograde translocation activity may explain the significant accumulation of ubiquitinated proteins in NSCLC (Figures 1 & 4a). We anticipate that the peri-nuclear cytosolic accumulation of ubiquitinated proteins (aggresomes) further stimulates VCP expression, leading to a chronic VCP induction (Figure 6). We designed this study to not only verify the elevated VCP protein expression in adeno- and squamous- NSCLC but also identify if it is involved in tumor pathogenesis and progression.

We performed a series of *in vitro* functional studies using VCP knockdown (RNAi) and a functional inhibitor (EerI) to assess the molecular links between chronic VCP induction and NSCLC pathogenesis. Our data suggest that VCP regulates a number of critical cellular processes involved in NSCLC tumor development, growth and metastasis. We found that inhibition of VCP protein expression or function reduced the proliferative capacity of NSCLC cells and induced apoptosis (Figure 2a & b), caused G0/G1-growth phase arrest (Figure 2c & d) and slowed NSCLC migration and invasion (Figure 3). Although we are the first to demonstrate the role of VCP in regulating key cellular processes in NSCLC, our findings are consistent with previous studies in yeast, *C. elegans* and non-neoplastic cells studying cell proliferation and apoptosis [1], [9]. In addition to proliferation and apoptosis, we found that VCP also mediates tumor cell migration and invasion suggesting its critical role in NSCLC pathogenesis and progression. Moreover, each of these processes is considered to be one of several key “hallmarks of cancer” – distinctive and complimentary capabilities that must be acquired by cells to enable tumor growth and metastasis [36]. Coupled with the evidence that VCP is essential for cancer cell survival (Figure 2), it is clearly an attractive molecular target for controlling tumor genesis and metastasis of NSCLC and other forms of cancers because its successful inhibition may control multiple critical stages involved in tumor formation and progression.

In order to determine the molecular mechanism by which elevated VCP expression promotes these various cellular processes involved in NSCLC pathogenesis and progression, we examined the expression the levels of several cancer-related proteins. We observed the significant induction of Nrf2 and SIRT1 and suppression of p53 in NSCLC cell lines (H1299 and H1944) as compared to control (Beas2b and HBE) cells (Figure 4a). Further experimental analysis revealed that VCP not only participates in the proteasomal degradation of tumor suppressor p53 but also forms a complex with p53 that can be detected by immunoprecipitation (Figure 4b & c). This finding confirms and adds to a previous study that suggested that RNAi of VCP may rescue p53 from proteasomal degradation in HeLa cells [37]. Moreover, our data suggests that VCP regulates p53 levels by proteasomal degradation pathway. Although, p53 is known to be degraded by the proteasomal pathway [38], [39] but we show here for the first time the critical intermediate involved in this process (Figure 4d). Further studies are required to identify the precise mechanism of VCP mediated p53 degradation. Nonetheless, we found here that VCP regulates protein levels of p53, the principal tumor suppressor that is a transcription factor capable of activating genes involved in cell cycle regulation, apoptosis and countless other processes [40], [41], [42]. This may also explain anti-proliferative and pro-apoptotic effects of VCP inhibition in NSCLC (Figures 2 & 6). We also confirm (Figure 4b) here the well-characterized role of VCP in regulating NFκB levels, a key metastasis mediator [9], [16]. Elevated VCP expression in NSCLC likely leads to increased proteasomal degradation of IκB, the endogenous inhibitor of NFκB, explaining NFκB-induced pro-survival/anti-apoptotic signaling. Recent evidence also suggested that VCP and/or its associated co-factors are also implicated in the direct regulation of HIF1α (hypoxia-inducible factor 1α; tumor angiogenesis and metastasis promoter) [43], Nrf2 [8] and Aurora B kinase (implicated in genomic instability) [44]. Clearly VCP inhibition can restore p53 and NFκB protein expression based on our data (Figures 4 & 6), indicating towards it therapeutic potential in treating tumor progression and metastasis.

In conclusion, we report for the first time a thorough analysis of the molecular links between elevated VCP protein expression and NSCLC pathogenesis and progression. VCP over expression may facilitate malignancy by promoting NSCLC cell growth, proliferation, migration and anti-apoptosis through abnormal expression of several cancer-related proteins, including p53 and NFκB. Moreover, we demonstrate the therapeutic potential of targeting VCP by a small-molecule functional inhibitor (EerI, Eeyarestatin I) using, *in vitro* and murine models (Figures 3d & 5). Recent studies have revealed that EerI binds to both VCP and the ER (endoplasmic reticulum) membrane and inhibits VCP-dependent protein degradation as well as VCP-associated deubiquitinating enzymes [18], [45]. We would like to emphasize that EerI or another potent VCP inhibitor may have added potential as a novel cancer therapeutic over existing proteostasis and HDAC (histone deacetylase) inhibitors [such as the FDA-approved drugs bortezomib/PS-341 [46] and SAHA [47]] as its targets upstream VCP-retrograde translocation step prior to proteasomal and HDAC6 mediated protein processing as we recently discussed [9], [35]. Based on the dual therapeutic potential of VCP inhibition as well as its ability to regulate critical oncogene and tumor suppressor protein levels, we are currently evaluating its therapeutic efficacy in controlling NSCLC metastasis using an intrapulmonary tumor implantation murine model.

## Supporting Information

Figure S1 **VCP regulates apoptosis of NSCLC.** TUNEL assay of H1299 cells transfected with pSM2 control or VCPshRNA (for 48 hrs) was used to identify number of apoptotic cells. Nuclear (blue, Hoescht) and TUNEL (green) staining of representative areas of each treatment is shown (white bar = 200 µm). We observed a 6-fold increase in the number of TUNEL-positive cells by VCP inhibition (shRNA-2&-3) as compared to control (pSM2). *VCP inhibition controls the tumorigenic capacity of NSCLC by inducing apoptosis.*
(TIF)

Methods S1 **Terminal Transferase dUTP Nick-End Labelling (TUNEL) Assay.**
(DOC)
