# Supplementary figures and images for: Critical Role of VCP/p97 in the Pathogenesis and Progression of Non-Small Cell Lung Carcinoma
Source: PLoS One. 2011 Dec 22;6(12):e29073. doi: 10.1371/journal.pone.0029073 (PMC3245239; doi:10.1371/journal.pone.0029073)

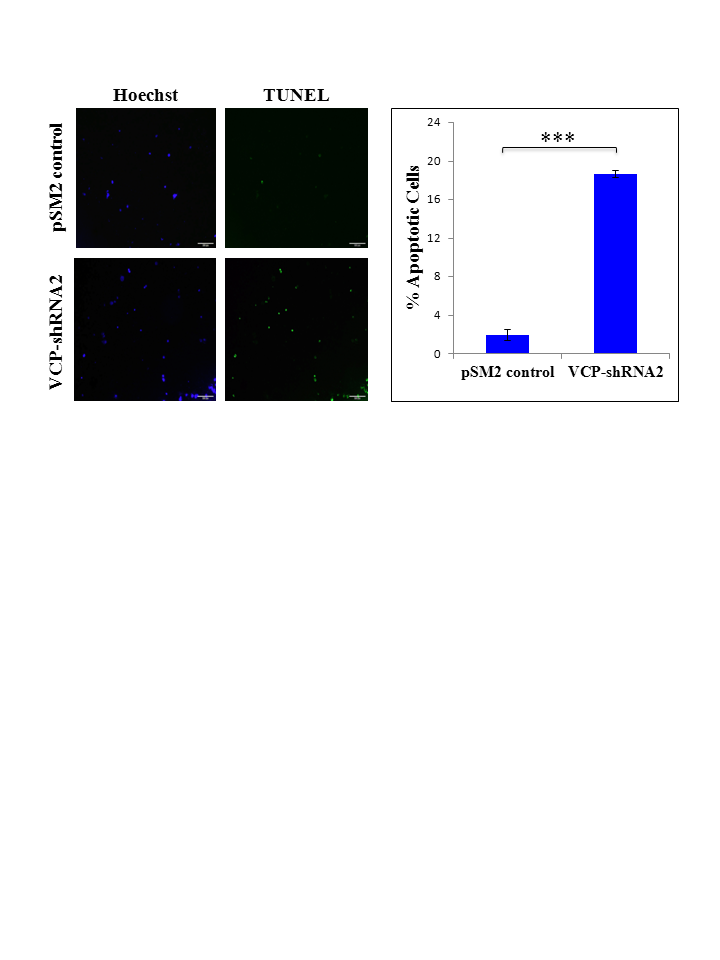

Supplement: Figure S1 — VCP regulates apoptosis of NSCLC. TUNEL assay of H1299 cells transfected with pSM2 control or VCPshRNA (for 48 hrs) was used to identify number of apoptotic cells. Nuclear (blue, Hoescht) and TUNEL (green) staining of representative areas of each treatment is shown (white bar = 200 µm). We observed a 6-fold increase in the number of TUNEL-positive cells by VCP inhibition (shRNA-2&-3) as compared to control (pSM2). VCP inhibition controls the tumorigenic capacity of NSCLC by inducing apoptosis. (TIF) [file pone.0029073.s001.tif]
