# Supplementary material for: Critical Role of VCP/p97 in the Pathogenesis and Progression of Non-Small Cell Lung Carcinoma
Source: PLoS One. 2011 Dec 22;6(12):e29073. doi: 10.1371/journal.pone.0029073 (PMC3245239; doi:10.1371/journal.pone.0029073)
Supplement: Methods S1 — Terminal Transferase dUTP Nick-End Labelling (TUNEL) Assay. (DOC) [file pone.0029073.s002.doc]

**Methods S1. Terminal Transferase dUTP Nick-End Labelling (TUNEL) Assay.** Apoptotic cells were identified using the DeadEnd Fluorometric TUNEL System (Promega) following the manufacturer’s instructions. To counterstain the nuclei, cells were incubated with 1μg/ml Hoechst (Invitrogen). Samples were analyzed under a fluorescence microscope using standard FITC and DAPI filter sets to view the flourescein (green) and the blue fluorescence of Hoechst (blue). Four fields with 100 cells each were counted randomly for each treatment and data on number of apoptotic cells (green) was summarized as mean ± SEM.
